# Therapeutic Strategies and New Intervention Points in Chronic Hepatitis Delta Virus Infection

**DOI:** 10.3390/ijms160819537

**Published:** 2015-08-18

**Authors:** Zhimin Guo, Thomas King

**Affiliations:** 1 3130 Huron Peak Ave., Superior, CO 80027, USA; E-Mail: Zhiminguogg@gmail.com; 2 Allevagen, LLC, 4105 Perry St., Denver, CO 80212, USA

**Keywords:** HDV, HBV, HIV, deltavirus, hepatitis, therapeutic, clinical, trial

## Abstract

Chronic hepatitis delta virus infection (CHD) is a condition arising from super-infection of hepatitis B virus (HBV)-infected patients, resulting in a more rapid advance in liver pathology and hepatocellular carcinoma than is observed for HBV mono-infection. Although hepatitis delta virus (HDV) is structurally simple, its life cycle involves the complex participation of host enzymes, HBV-derived surface antigen (HBsAg), and HDV-auto-ribozyme and hepatitis delta antigen (HDAg) activities. Unsatisfactory clinical trial results with interferon-based therapies are motivating researchers to adjust and redirect the approach to CHD drug development. This new effort will likely require additional structural and functional studies of the viral and cellular/host components involved in the HDV replication cycle. This review highlights recent work aimed at new drug interventions for CHD, with interpretation of key pre-clinical- and clinical trial outcomes and a discussion of promising new technological approaches to antiviral drug design.

## 1. Introduction

Hepatitis delta virus is a defective RNA virus whose propagation requires the support of surface protein (HBsAg) of HBV and can occur either as a co-infection with HBV or by superinfection of chronic hepatitis B (CHB) patients [1]. It is the latter that most often leads to chronic HDV infection (CHD), a condition accompanied by exacerbation of pre-existing HBV-induced liver damage and much greater risk of cirrhosis, hepatic decompensation, hepatocellular carcinoma and death [2]. Dual infection of HBV/HDV is linked to a 10 year mortality rate of 16%–20%, significantly higher than is observed for HBV monoinfection [1,3].

An estimated 15 to 20 million people worldwide are infected with HDV and although the incidence of CHD is decreasing in some of the more industrialized nations due to improved preventative measures, this is not the case in all developed regions [4], and the disease remains a significant public health concern in developing regions including much of Asia and the pacific islands [5]. Importantly, the rate of HDV infection in HIV/HBV-coinfected patients has increased notably since 2001 with a ~20% HDV infection rate in this population [6]. HDV is also growing in prevalence among intravenous drug users [7].

In light of these statistics, it is obvious that improved CHD therapies are required but the disease has not ranked highly as a commercial target for drug development companies, probably because the more highly affected regions occur in developing countries without robust health insurance infrastructures. Nevertheless, clinical development efforts have been in progress since the early 1990s testing various interferon-based regimens with and without nucleoside-based HBV polymerase inhibitors in CHD subjects. Low response rates have thus far been observed in IFN-treated CHD patients (~20%–43%) after a 12 to 24 month (or longer) treatment courses with high-dose interferon, and the well-characterized side effects of these regimens are an obvious concern. HBV-targeted nucleos(t)ide analogs and related compounds including Ribavirin, Adefovir, Tenofovir, Famciclovir, and Lamivudine have been tested as standalone treatments in CHD patients, but somewhat surprisingly, no significant virologic improvements were observed in these trials [8]. This reflects that these compounds do not completely eradiate the virus and HDV can replicate with small amounts of bioavailable HBsAg.

Despite these challenges, a strong knowledge of the general biology and replication mechanism of HDV has identified a number of new intervention points that could be exploited to block one or more steps in the viral life cycle. This article provides an update on the latest advances in our understanding of HDV biology and provides a summary of therapies that have been or are currently being pursued in the clinic. We also review emergent approaches that are in the pre-clinical testing phase and these are discussed with a special eye on the strengths of the various intervention points. Detailed epidemiological data and advances in diagnostic procedures are discussed in other excellent reviews [5,9,10].

## 2. HDV Biology

### 2.1. HDV Structure

Hepatitis D virus is the smallest known human pathogen, a 36 nm roughly spherical virus composed of a single stranded RNA genome bound to the hepatitis delta antigen (HDAg) that is surrounded by a HBsAg coat. The HDV genome is a single stranded ~1.7 kb circular RNA complexed with ~70 molecules of a mixture of short (195 amino acids) and long (214 amino acids) forms of HDAg [11]. These variants are produced from the same gene as a result of a RNA editing at the stop codon of the shorter HDAg ([12] see below). HDAg dimerizes through an antiparallel coiled-coil and these dimers then associate further into octamers that form a 50 angstrom hole lined with basic amino acid side-chains [13]. This latter structure has been proposed to form a clamp around the RNA, by structural analogy to other DNA and RNA clamping proteins [13].

The HDV core RNP is surrounded by ~100 molecules of HBsAg (small, medium and large variants combined) in association with host membrane-derived lipids. The S-HBsAg component mediates HDV virion particle formation, while L-HBsAg is essential for entry, assembly and infectivity of the virus [14,15,16].

### 2.2. HDV Life Cycle

**Figure 1:**
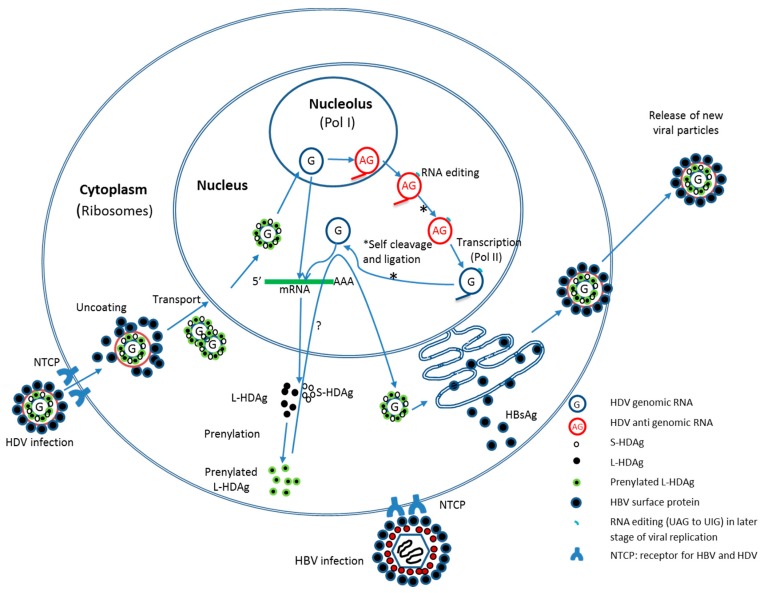
Model of the hepatitis delta virus (HDV) life cycle.

While the composition of HDV is remarkably simple by comparison to many other human viral pathogens, its life cycle is equally complex. The entry of HDV into hepatocytes is considered to be similar to HBV and begins with binding to heparan sulfate proteoglycans on hepatocyte surface. A recently discovered membrane protein known as sodium taurocholate co-transporting polypeptide (NTCP) is believed to be the key receptor for entry of both HBV and HDV into hepatocytes and the pre-S1 domain of L-HBsAg is a required determinant for association [16,17,18,19]. Once HDV gains cellular entry, the particle is uncoated and the viral genome is translocated by HDAg into the hepatocyte nucleus. The genomic RNA is then replicated by a double rolling circle mechanism by RNA polymerase I (presumably in the nucleolus [20]) to generate an oversized complementary RNA that is cleaved to the correct length by the self-encoded ribozyme, and ligated by a host cellular enzyme to form a circular RNA antigenome. A functional genome is then produced from the antigenome via this same cycle of rolling circle replication/cleavage-ligation except that replication in this case is catalyzed in the nucleoplasm by host RNA polymerase II. Functional 5′ capped and 3′ polyadenylated mRNAs copies are produced from the genomic RNA by RNA polymerase II and exported to the cytoplasm for translation (Figure 1) [5,20,21].

A unique facet of HDAg expression is the production of two different forms of the antigen from the same coding region. An RNA editing event occurs on the antigenomic (AG) RNA during replication, that is catalyzed by a host adenosine deaminase (ADAR1) and this changes a UAG stop codon in S-HDAg to a UGG (tryptophan), resulting in a C-terminally extended variant (L-HDAg) that is 19 residues longer than S-HDAg [22,23]. ADAR1 (Adenosine deaminase acting on RNA 1) catalyzes the conversion of adenosine to inosine and the latter is translated by the cell as a guanosine [24]. The ADAR1- catalyzed reaction of the HDV AG could be an interesting target for therapeutic intervention (see below). The additional C-terminal residues of L-HDAg that result from the editing confer its functional properties that differ from those of S-HDAg; whereas S-HDAg is required for the initiation of the viral genome replication, L-HDAg serves as a key inhibitor of replication and is essential for the assembly of new virion particles. Thus, the RNA editing activity resulting in the UAG→UGG read-through mutation serves a central function in the HDV replication cycle [25].

In addition to this post-transcriptional point of regulation, post-translational effects also play a role in HDV life cycle. In the presence of HBsAg, L-HDAg localizes to the cytoplasm where post translational modifications occur and these are important for HDV replication and virion assembly. Isoprenylation, phosphorylation, methylation, acetylation and sumoylation are all found to occur in the HDAg. Isoprenylation of L-HDAg enhances its inhibition of replication and is also required for viral particle formation because prenylation of L-HDAg mediates its association with HBsAg (this fact has been exploited by drug developers, see below) [26]. Phosphorylation and methylation of S-HDAg are important for RNA replication by regulating its RNA-binding activity, and acetylation of lysine 72 of S-HDAg influences its cellular localization and RNA replication. Lastly, sumoylation (addition of small ubiquitin-like modifiers) of S-HDAg increases HDV genomic RNA and mRNA synthesis [27].

### 2.3. HDV Pathogenesis

HDV replication occurs exclusively within hepatocytes and its pathological effects are believed to be localized to the liver. Several distinct mechanisms are thought to be responsible for HDV-induced the liver damage. The first but more controversial is a direct cytopathic effect found mainly in acute HDV infection. Taylor *et al* showed that high levels of the HDAg and viral RNA caused G1 phase cell cycle arrest [28]. Other effects presumably related to this and driven mainly by S-HDAg expression include shrunken eosinoiphilic cytoplasm and pyknotic nuclei [29]. A second and more thoroughly characterized root cause of the pathology in CHD is traced to immunological effects such as: (i) excessive recruitment of inflammatory cells due to NFkb upregulation, resulting in the production of TNFα and other innate cytokines; (ii) inhibition of type 1 interferon (T1IFN) signaling, and; (iii) HDV-specific Th1 CD4- and CD8 cytotoxic T cell responses, which although likely beneficial for the ability to clear HDV infected cells cause tissue damage if proper immune regulation (e.g., PD-1) is absent or insufficient. The second point is worth more discussion here, because the anti-IFN-α/β response may be a major factor for immune evasion in CHD, as it is in other viral diseases including HCV and influenza.

IFN-α/β responses are initiated in virus-infected cells when double-stranded RNA (dsRNA) intermediates are released following viral entry. The transcription factors NF-κB, interferon regulatory factor (IRF)-3 and 7, and activating transcription factor 2 (ATF2)/c-Jun are activated and induce expression of the alpha and beta IFNs. These in turn bind to the IFNα receptor (IFNAR) of infected cells, resulting in activation of the JAK-STAT pathway and transcription of >100 genes whose collective effect is to establish an antiviral state [19,30]. The apparent ability of HDV to circumvent this powerful antiviral response stems in part from that its genome is single stranded RNA, whereas the most efficient induction of the IFN-α/β response occurs through dsRNA-dependent activation of TLR3 [31]. HDV also avoids the effects of T1IFN by blocking Tyk2 activation, selectively impairing the activation and nuclear translocation of STAT1 and STAT2 [32]. The dampened (or absent) IFN-α/β transcriptional response to HDV infection is a common feature other viruses in this family as well, including woodchuck hepatitis virus [33]. It is noted, however, that in a very recent study with HDV-infected NTCP-expressing mice, the IFN-α/β response pathway was activated by HDV infection as measured by the induction of interferon response genes (ISGs)—an effect that required expression of the IFN α/β receptor (IFNα/βR1 −/−). Interestingly, in IFNα/βR1 −/− mice, the efficiency of HDV infection was ~10 fold higher than in wild type IFN α/β receptor mice, but infection was also eliminated very efficiently, indicating that clearance can occur via a type-I interferon independent pathway [19]. Thus, the involvement of the type I IFN pathway in the host response to HDV appears to be complicated and the species and experimental systems used to study it may strongly affect the outcomes. Further studies in this fascinating aspect of anti-HDV immunity will hopefully converge on a deeper understanding of the role ISGs play in CHD.

The proposed role of HDV in tumorigenesis in hepatocellular carcinoma was the topic of recent detailed studies. One article reported that the production of reactive oxygen species was elevated in L-HDAg-expressing cells and that STAT-3 and NF-kB were up-regulated via the oxidative stress pathway. These factors are hypothesized to result in enhanced cell survival, fibrogenesis, cell proliferation and angiogensis—all features linked to tumor formation and/or maintenance [34].

In a second mechanism, HDV increased the expression of clusterin protein by enhancing the acetylation of histone H3 protein in the region of the clusterin promoter. The increased clusterin levels were reported to play a significant role in tumor initiation [35,36]. In a more recent but entirely *in vitro* study, a mass spectrometry based quantitative proteomics approach identified a strong positive relationship between HDV replication and expression of genes linked to tumorigenesis [37]. Increased expression of pro-inflammatory growth and anti-apoptotic factors largely explain the pronounced necrotic and inflammatory cycles characterized by CHD. These processes ultimately lead to early progression to hepatocellular carcinoma via oxidative stress—induced aberrant signaling pathways in HDV patients. 

### 2.4. Therapeutic Strategies in CHD

Acute co-infection of HDV and HBV is clinically indistinguishable from acute hepatitis B. Both viruses are usually cleared, with less than 5% of co-infected patients progressing to chronicity. Treatment of acute co-infection of HDV/HBV is same as the treatment of acute hepatitis B and is mainly supportive [1]. HDV superinfection of chronic HBV patients will progress to chronicity in most (80%–90%) patients, and here we review CHD-focused treatment strategies.

Although commercial interest in developing better ways to attack CHD has been historically limited, this situation is changing. HBV remains uncured, immigration is contributing to viral spread, and improved diagnostics and emerging epidemiological data more clearly illuminate the depth of this unmet need.

The primary challenges in developing HDV therapies include that: (i) HDV encodes only one protein and is highly dependent on the host machinery for replication, translating to limited opportunities for virus-targeted approaches (although host enzyme targeting is gaining momentum in antiviral research), and; (ii) the virus can replicate with a very small supply of HBsAg. Current HBV-antivirals do not eradicate HBsAg and thus do not control HDV [38].

The prevailing approach to drug development thus far has been to identify previously approved drugs that have a mid- to high level of viral suppressive activity in HBV and to evaluate these in CHD patients.

#### 2.4.1. Immunomodulatory Drugs

As the interferons have a long history of clinical use and are somewhat active in CHB patients it was logical to try these compounds in CHD. Numerous controlled clinical trials have been conducted since the early 1990s testing various interferon-containing treatment regimens in CHD subjects [39]. Despite these efforts, disappointing response rates were generally observed (~up to 43%) after a 1 to 2 year treatment course [40,41,42,43]. At least one large observational retrospective study is also closely aligned with this conclusion [43]. The interferons are unfortunately poorly tolerated and show high relapse rates of 60%–97%. More recently, however, clinical data have indicated that pegylated IFN alpha (PEG-IFNα) is better tolerated and patients may be dosed continuously for up to 96 weeks. A study testing the effect extended peg-IFNα dosing was therefore conducted in 13 advanced disease CHD patients, with continuous safety monitoring and dose adjustment as needed to ensure patient safety (Table 1)] [44]. The primary histological endpoint of the study was improved hepatic histology following 144 weeks of therapy. The primary virological endpoint was defined as a complete virologic response (CVR)—a combination of reducing serum HDV RNA to below detection plus attainment of HBsAg seroconversion. Due to serious IFN-related adverse events in a few of the 13 patients, not all of the subjects were evaluated. Of the 12 patients treated for at least 6 months, 3 achieved a CVR after 24, 37, or 202 weeks of treatment (25%, consistent with the summary described for other IFN treatments above). Two additional patients became HDV RNA negative but subsequently showed on-treatment virologic breakthrough. Histological improvements were observed in some patients although the results were not statistically significant. The authors concluded that despite the increased doses and long treatment duration, PEG-IFNα remains unsatisfactory for the treatment of CHD. In what could be a final wave of IFN trials, combinations of nucleos(t)ide inhibitors with PEG-interferon are under evaluation in well-controlled phase 2 and 3 studies (Table 1) and it will be of great interest to determine if further gains in activity can be achieved by concomitant immunomodulation and polymerase inhibition.

An interesting case report illustrates that under the right conditions this drug combination does have potential. A patient initially presenting with high serum HBV DNA (>9 log UI/mL) and with HDV-RNA of up to 5.6 log (copies/mL) achieved complete resolution of HBV-HDV co-infection following treatment with the following regimen. First, the patient was treated for 2 months with 180 µg/week of Pegasys which had no apparent effect on HBV DNA, and HDV RNA was still detected. Then, tenofovir disoproxil fumarate (TDF; Viread) was administered with Pegasys at 300 mg/day for an additional 5 months, causing a 7-log drop in HBV DNA and disappearance HDV RNA. Due to side effects the Pegasys dose was then reduced to 135 µg/week and the reverse transcriptase inhibitor emtricitabine (a cytidine analog) was added to the regimen for 4 additional months. This led to complete resolution of the HBV-HDV co-infection and the patient remained negative for HBV DNA, HBsAg and HDV RNA at one year post-treatment [45]. The success story illustrates the concept that the right combination of immunomodulation and polymerase inhibition can be effective, with continued adjustment of the treatment regimen as patient parameters evolve. The challenge will be to identify the ideal dose and timing needed to achieve activity in most subjects, and the degree to which this is influenced by pre-treatment HBV and HDV viral loads. This last point will be of growing importance as effective new therapies emerge and their regimens optimized. Most HDV-positive patients have very low serum HBV DNA, but some do exhibit signs of very active HBV replication, with HBeAg positive status and high viral HBV DNA titers [46]. Understanding how HBV viral load should direct HDV treatment is an important goal and data from ongoing clinical trials of new therapies (see below) should eventually help clinicians address this.

#### 2.4.2. HBV Polymerase Inhibition as Standalone Therapy

Nucleot(s)ide analogs and related compounds targeting HBV have been tested in CHD patients, including Ribavirin, Adefovir, Famciclovir, and Lamivudine but no striking virologic improvements were observed in these trials [8]. For example, the end of follow-up virologic response to Lamivudine (6 month SVR) in one study was 11%–13% [39]. As discussed above, these facts probably reflect that the compounds do not completely eradiate HBsAg.

#### 2.4.3. Prenylation Inhibitors

Eiger Biopharmaceuticals is investigating the safety and efficacy of prenylation inhibitors in CHD infection. Isoprenylation of L-HDAg enhances its inhibitory effect on HDV genome replication and more importantly is required for viral particle formation because this modification mediates association of L-HDAg with HBsAg. The prenylation inhibitor Lonafarnib is being tested in phase 2 trials in combination with Ritonavir or with both Ritonavir and PEG-IFN. Ritonavir “boosting” is best known for its ability to enhance the pharmacokinetics of protease inhibitor cocktails in HIV treatment regimens, by inhibiting the key drug metabolizing enzyme cytochrome P450 and an efflux transporter that would otherwise pump the drugs out of the gut wall and back into the intestinal lumen [47]. We were unable to locate peer reviewed publications of phase I trial results with prenylation inhibitors such as Lonafarnib, but the addition of Ritonavir in phase 2 trials may reflect sub-par pharmacodynamics of standalone Lonafarnib in CHD. A publication of phase 2A clinical trial results by Eiger indicates that up to a 1.4 log IU/mL decline in viral load was observed in CHD patients treated with 200 mg twice daily for 28 days with 6 months follow-up [48]; these results were without Ritonavir boosting and further gains in efficacy may be observed with Ritonavir. The U.S. Food and Drug Administration has granted fast track designation for Lorafarnib treatment in this indication. These data reflect a predicted outcome based on promising murine efficacy studies of prenylation inhibitors some 12 years ago [49]. Although early studies with Lorafarnib revealed gastrointestinal toxicities and myelosuppression and/or encephalopathy in some patient populations [50], these compounds were reasonably well-tolerated in a variety of cancers in some cases in combination with chemotherapeutic agents [51].

**Table 1:** Current or recently completed clinical trials testing therapies for chronic hepatitis delta (CHD) infection. Abbreviations: Immunomod: immunomodulatory mechanism; NAP: Nucleic Acid Polymer; CYP3A4, Cytochrome P450 3A4; NA, not applicable. Eiger: Eiger Biopharmaceuticals.

Compound(s) Tested/Class	Class	Phase of Trial (n)	Sponsor	Status as of this Writing	Publication/Observations
(1) EBP921	prenylation inhibitor	Phase 1 (8)	Eiger	Unknown	NA
(1) Lonafarnib	(1) farnesyl transferase inhibitor	Phase 2 (40)	Eiger	Recruiting	NA
(2) Ritonavir	(2) inhibitor of HIV protease and CYP3A4				
(1) Lonafarnib	(1) see above	Phase 2 (21)	Eiger	Recruitiung	Ritonavir + lonafarnib: 3.2 log_10_ decline in viral load was observed at week 8 of treatment
(2) Ritonavir	(2) see above				
(3) Peginterferon	(3) immunomod				
(1) Peginterferon alpha2A	(1) immunomod	Phase 2 (13)	NIDDK	Completed (results published)	Heller, Rotman *et al.* (2014) [44]
(1) REP 2139-Ca	(1) NAP:blocks HBV subviral particle formation	Phase 2 (12)	REPLIcor	Ongoing (not recruiting) mid 2016 estimated completion	~6 log_10_ decline HDV RNA, standalone REP2139-Ca
(2) Pegasys^®^	(2) immunomod				
(1) Ribavirin	(1) nucleoside inhibitor	Phase 4 (20)	National Taiwan Univer. Hospital	Unknown	NA
(2) Pegylated Interferon Alfa-2B	(2) immunomod				
(1) Peginterferon alpha2A	(1) immunomod	Observational	Hoffman LaRoche	Ongoing, not recruiting	NA
(1) Peginterferon alpha2A	(1) immunomod	Phase 2 (70)	HepNet Study house; Hoffman La-Roche; Gilead	Ongoing, not recruiting	NA
(2) Tenofovir	(2) nucleotide analog				
(1) Lonafarnib	(1) farnesyl transferase inhibitor	Phase 2 (14)	NIDDK	Completed	NA
(1) Peginterferon alpha2A	(1) immunomod	Phase 3 (50)	Hoffman-La Roche	Ongoing, not recruiting	NA
(2) Tenofovir	(2) nucleotide analog				
(1) Myrcludex B	(1) hepatocyte entry	Phase 2a (24)	Hepatera	Completed	Myr standalone: >1Log_10_ HDV RNA decline and ALT normalization in 4 subjects at week 24
2) PEG-IFNa	(2) immunomod				

#### 2.4.4. RNA Interference

It is well-known that the liver efficiently accumulates nucleic acid-based therapeutics including antisense oligonucleotides and siRNAs [52,53]. This is underscored by an FDA approval of an antisense oligo for the treatment of familial hypercholesterolemia and a current phase 3 clinical trial by Alnylam, Inc. for the treatment of TTR (Transthyretin)—Mediated Amyloidosis (see [54] for phase 1 Proof-of concept data) [55]. Preclinical studies in mice showed that with the appropriate cellular targeting, a hepatocyte-targeted cholesterol-conjugated siRNA recognizing conserved HBV sequences resulted in a multi-log reduction in viral load [56]. This and related studies paved the way for the inevitable testing of siRNA therapies for CHD [57]. Alnylam announced a clinical-track program to develop siRNA cocktails targeting both HBV RNA and the HDV genome, an approach that could have powerful therapeutic effects in man if and only if a well-tolerated regimen can be identified. Nucleic acid therapeutic human trials have been substantially challenged by toxicities but recent changes to nucleotide chemistry and route of administration (subcutaneous) are improving the therapeutic indicies [52].

#### 2.4.5. Nucleic Acid Polymers

Nucleic acid polymers (NAPs) cleared serum HBsAg pre-clinically in HBV-infected ducks and acts synergistically with pegylated IFNα-2a and thymosin α-1 to restore host immunological control of HBV infection [58]. A NAP named REP 2139-A is proposed to prevent formation of HBsAg subviral particles thereby unmasking an underlying pre-existing anti-HBsAg (anti-HBs) response. This compound is currently being tested in a small phase 2 trial in combination with Pegasys (Table 1; REPLIcor). As with other nucleic acid therapies and especially phosphorothioate-based chemistries, toxicity can be a real problem so finding a well-tolerated dose and treatment duration for these compounds will be key deliverables for the platform. Results were recently presented at the EASL 2015 conference in which REP 2139 (Table 1) induced a ~4 log reduction in serum HDV RNA and serum HBsAg in a small phase 2 trial with CHD patients (Table 1). It will be exciting to view these results when they are formally published.

#### 2.4.6. Therapeutic Vaccines

A therapeutic vaccine capable of mounting efficient CD4^+^ and CD8^+^ T responses recognizing HBV and/or HDV antigens could be a promising approach for CHD therapy, if clearance of infected cells can be elicited in the suboptimal immunological background described above. Several efforts demonstrated preclinical and clinical immunogenicity of T cell-inducing HBV vaccines in murine and woodchuck models for HBV [38,59] and in CHB patients [60]. Immunization with HDV antigen-containing DNA vaccines does generate Th1 T cell responses and a HDV p27 DNA vaccine/adenoviral prime-boost vaccination regimen protected woodchucks from simultaneous WHV/HDV challenge in 5 of 7 animals [61,62,63,64]. In the woodchuck study [61], serum HDV RNA could not be detected in the 5 protected animals throughout the monitoring period (weeks 1–19 post-challenge) whereas the two woodchucks that were not protected by vaccination showed peak HDV RNA levels of up to 10^10^ copies/mL. Such dichotomous outcomes with small cohorts are not surprising given that woodchucks are outbred. Interestingly, however, a recent study showed that intrahepatic clearance of HDV can be achieved in severe combined immunodeficiency (SCID) mice, suggesting that adaptive T cell-mediated immune responses are not required for viral elimination and innate immunity may play an even bigger role than originally thought [19]. It will be of interest to determine if this is an anomaly linked to the infectious murine model or if the behavior is more universal. Supporting the latter idea, recent work on the innate response to HDV/HBV co-infection in humanized mice showed a remarkably strong induction of innate immune responses as compared to the response in HBV mono-infected mice [65]. Further work in this promising area is justified given recent preclinical successes of HBV and woodchuck hepatitis virus (WHV)-targeted therapeutic vaccines [38,59,66]. Combination therapies featuring vaccine plus direct anti-HDV compounds may be a particularly promising strategy.

#### 2.4.7. Blocking Viral Entry

A compound known as Myrcludex B blocks NTCP, the receptor essential for both HBV and HDV entry into hepatocytes. This compound is a *N*-acylated preS1-derived lipopeptide that inhibits HBV and HDV entry *in vitro* and *in vivo* with high efficacy [67]. Results from a phase 2a trial (*n* = 24) testing Myrcludex B alone and in combination with PEG-IFNα indicated that Myrcludex was well-tolerated both as monotherapy and in the combination regimen. Six of seven evaluable patients in the Myrcludex B monotherapy cohort showed >1 log10 HDV RNA decline, and ALT normalization occurred in 4 subjects at week 24. In the combination arm, all patients showed HDV RNA decline and 5 were HDV RNA negative at week 24 [68]. The Myrcludex monotherapy results are promising for a first phase 2 trial, given that the drug’s mechanism of action is presumably limited to preventing infection of uninfected hepatocytes and the agent is not expected to root out or eliminate previously infected hepatocytes.

#### 2.4.8. Other Points of Intervention

Additional points of therapeutic intervention for CHD include interference with key post-transcriptional and post-translational modifications to HDV RNA and proteins. An interesting example of this would be inhibition of the ADAR1-mediated RNA editing event that is required for forming the large isoform HDAg. The apparent lack of focus on developing small molecule inhibitors of ADAR1 is probably explained by the observation that mice lacking the gene do not survive beyond 12 days post-coitus and display severe defects in liver development, suggesting critical functions for the human ortholog [69]. An attractive alternative could be to block the substrate rather than inhibiting the ADAR1 enzyme itself, avoiding potential toxicities that could otherwise result from inhibiting other cellular targets of this enzyme. A similar substrate-blocking principle or even small molecule inhibitor strategies could be optimized to inhibit the ribozyme-mediated cleavage that is required for proper genomic processing. A large body of work has been published that delineates the mechanism and *cis*-acting sequences required for this cleavage reaction, forming a basis upon which to model inhibitor strategies [70]. The copper-complexed forms of several antibiotics (Amoxicillin, Apramycin, and Ristomycin A) cause strong inhibition of the HDV ribozyme *in vitro*, and while relatively high concentrations of these drugs were needed to achieve this effect (mid micromolar) the potential exists for structural modifications that could elicit more potent inhibition [71]. Lastly, as further structural and functional information more clearly assigns roles of other HDAg modifications in the replication cycle (e.g., sumomylation, phosphorylation, andmethylation), the feasibility of intervention strategies targeting these reactions can be more clearly assessed [27,72].

## 3. Conclusions

HDV superinfection of hepatitis B virus (HBV) carriers causes the most severe form of hepatitis in humans with significantly more rapid progression to Cirrhosis, HCC and death than is reported for HBV monoinfection [1]. With 15–20 million patients infected worldwide, the relatively limited focus on finding effective new therapies somewhat surprising, and may reflect some combination of: (i) the commercial challenges inherent in developing world disease therapies, and; (ii) a view that increased rates of HBV vaccination and a predicted HBV curative drug will lead to rapid reductions in CHD prevalence. The time required for the latter to reach fruition will certainly influence the resources diverted for CHD therapy research in the coming decade. Meanwhile, an evaluation of HDV epidemiology indicates that insufficient diagnostic standardization combined with increases in immigration has likely led to an underestimation of the true global prevalence [10].

Despite this assessment, diligent efforts have (and are being) made to thoroughly test interferon-based regimens alone and in combination with direct acting antiviral compounds. These studies showed the best efficacy to date with up to a 43% rate of sustained virologic responses (although the endpointsused in various studies are different). An analysis of the data suggests that a plateau of efficacy will soonbe reached with interferon-based regimens, and the unwanted side effects of these compounds haunt the industry.

The structural simplicity of HDV has led many to view the virus as essentially undruggable, but its life cycle presents several intervention points that have been explored minimally or not at all. One exception to this is the recent evaluation of prenylation inhibitors which appear to be making headway in this disease. More knowledge about the structure, function, and immunogenicity of the HDAgs and host-pathogen interactions should spur the discovery of additional effective new drugs and vaccines in the coming years.
